# Localization and segmentation of optic disc in retinal images using circular Hough transform and grow-cut algorithm

**DOI:** 10.7717/peerj.2003

**Published:** 2016-05-10

**Authors:** Muhammad Abdullah, Muhammad Moazam Fraz, Sarah A. Barman

**Affiliations:** 1 School of Electrical Engineering and Computer Science, National University of Sciences and Technology, Islamabad, Pakistan; 2 Faculty of Science Engineering and Computing, Kingston University, London, United Kingdom

**Keywords:** Optic disc, Retinal image analysis, Growcut algorithm, Glaucoma detection, Image analysis

## Abstract

Automated retinal image analysis has been emerging as an important diagnostic tool for early detection of eye-related diseases such as glaucoma and diabetic retinopathy. In this paper, we have presented a robust methodology for optic disc detection and boundary segmentation, which can be seen as the preliminary step in the development of a computer-assisted diagnostic system for glaucoma in retinal images. The proposed method is based on morphological operations, the circular Hough transform and the grow-cut algorithm. The morphological operators are used to enhance the optic disc and remove the retinal vasculature and other pathologies. The optic disc center is approximated using the circular Hough transform, and the grow-cut algorithm is employed to precisely segment the optic disc boundary. The method is quantitatively evaluated on five publicly available retinal image databases DRIVE, DIARETDB1, CHASE_DB1, DRIONS-DB, Messidor and one local Shifa Hospital Database. The method achieves an optic disc detection success rate of 100% for these databases with the exception of 99.09% and 99.25% for the DRIONS-DB, Messidor, and ONHSD databases, respectively. The optic disc boundary detection achieved an average spatial overlap of 78.6%, 85.12%, 83.23%, 85.1%, 87.93%, 80.1%, and 86.1%, respectively, for these databases. This unique method has shown significant improvement over existing methods in terms of detection and boundary extraction of the optic disc.

## Introduction

**Figure 1:**
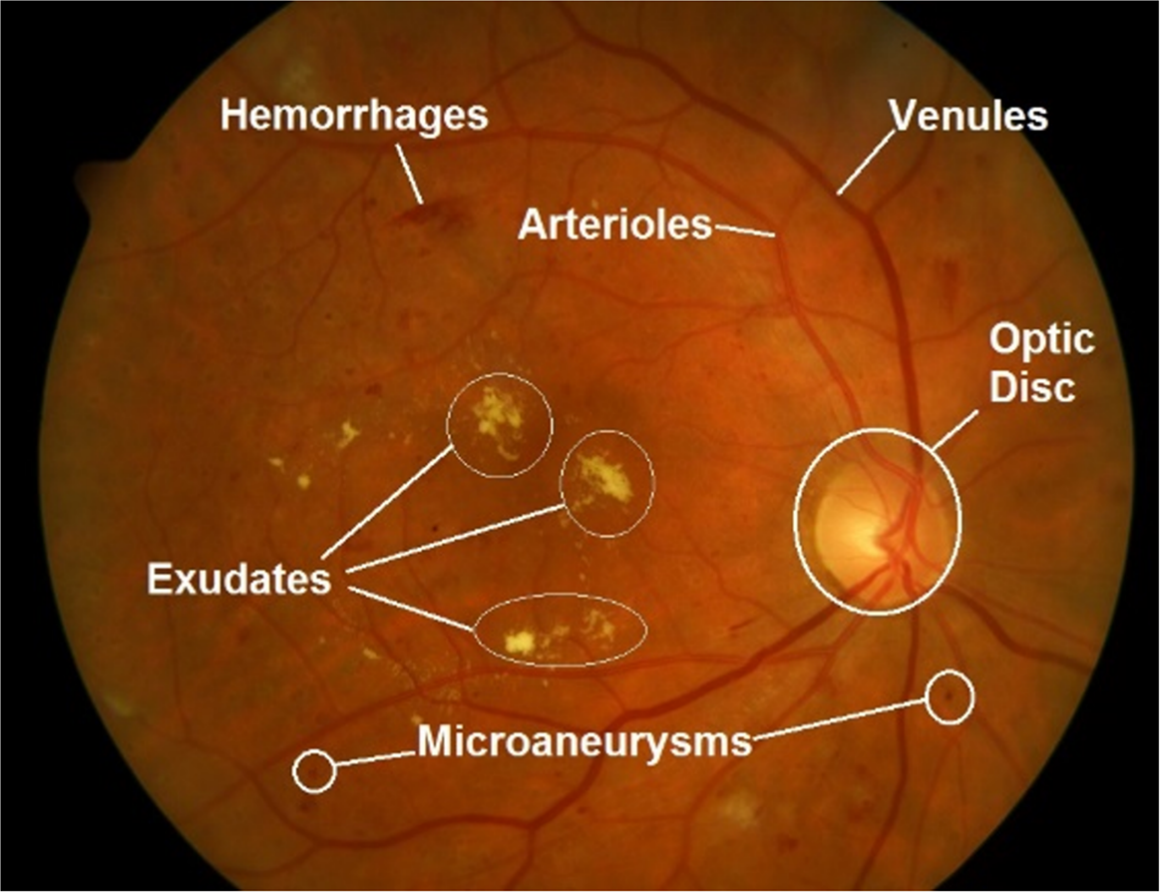
Important features in retinal fundus image.

Digital retinal images are widely used for early detection of retinal, ophthalmic and systemic diseases because they provide a non-invasive window to the human circularity system and associated pathologies (Jack & Brad, 2015). Glaucoma and diabetic retinopathy (DR) are among the major retinal diseases which are the leading cause of vision loss and blindness in the working population (Federation, 2013). Early detection of these disease by screening programs and subsequent treatment can prevent blindness. Computer aided diagnostic retinal image analysis is the first step in automated screening of these diseases in large population based studies (Fraz et al., 2015). The change in anatomical structures in human retina, which includes retinal vasculature, optic disc (OD), optic cup and retinal pathologies are the early diagnostic indicators of several diseases such as DR, macula edema and glaucoma (Jack & Brad, 2015). Among these, the OD is the most important feature because its visual aspects are central for glaucoma detection and other lesions assessment related to DR. The important anatomical structures presented in the retinal image are shown in Fig. 1. OD detection is preliminary step for glaucoma screening, which is globally the second leading cause of blindness. Moreover, it helps in the detection and localization of other retinal structures which includes the fovea, macula and estimating vascular changes (Basit & Fraz, 2015).

Glaucoma is caused by the increase in the intraocular fluid pressure in the optic nerve head (ONH), because of either blockage or a higher production of aqueous humor of the eye (Jack & Brad, 2015). Glaucoma remains asymptomatic at an early stage and slowly progress with time which ultimately leads to blindness. Medical treatment is only effective at the early stages because the optic nerve, once damaged can’t be cured (Weinreb, Aung & Medeiros, 2014). The early prevalence of glaucoma can be identified by localization and segmentation of the OD and optic cup, followed by computing the cup-to-disc ratio. The structural changes in OD furnish critical clues pertaining to glaucoma prognosis (Abramoff, Garvin & Sonka, 2010). A computer assisted diagnosis (CAD) system is necessary for large population based screening of glaucoma. OD localization and segmentation is the first step towards the development of CAD. Knowing the significance such systems, several OD approaches have been proposed and attempted by many but it’s still an active research area.

The OD appears as a variable sized, bright yellowish region, slightly oval in shape with blood vessels converging towards its center. These features are mostly used for automated OD localization. Retinal pathologies like exudates and lesions, if present, may appear like the shape of a disc, thus may cause false detection (Basit & Fraz, 2015). In CAD, accurate detection and segmentation of the OD is quite a challenging task because of various factors like boundary artifacts, missing edges and poor textural contrast. The variation in illumination conditions, luminosity and contrast during image acquisition are added challenges (Haleem et al., 2013). Moreover, the OD boundary is not constant because of the presence of incoming blood vessels which produce a fused boundary. Another distractor is papillary atrophy which, if present, appears as the bright region outside the OD and thus deforms the OD boundary (Aquino, Gegúndez-Arias & Marín, 2010).

This paper presents a new approach for automatic detection and segmentation of the OD based on morphological operations, circular Hough transform (CHT) (Illingworth & Kittler, 1987) and grow-cut (GC) algorithm (Vezhnevets & Konouchine, 2005). The GC algorithm has been widely used in many application areas of image segmentation, but has not been applied within the framework of retinal image analysis. To the limit of our knowledge, the GC algorithm has been utilized for the first time in localizing and segmenting the OD in retinal images. The method is evaluated on six retinal image datasets exhibiting different morphological characteristics. Experimental evaluation shows that this method is computationally fast in processing, robust to the variation in image contrast, illumination and presence of pathologies; and comparable with the state-of-the-art methodologies in terms of quantitative performance metrics.

It’s worth mentioning that this work is aimed at contributing to the development of automatic systems for glaucoma detection that are currently under development. Although other published solutions can be used, this work presents higher accuracy, robustness and is tolerant to a vast variety of image characteristics, which make it suitable for integration with a glaucoma detection system.

The organization of the paper is as follows. ‘Related Work’ presents a comprehensive overview of the OD localization and segmentation methodologies available in the literature. ‘The Methodology’ explains the proposed methodology in detail. The materials and the performance metrics used to evaluate the proposed methodology are illustrated in ‘Material.’ The results and comparison with other methods are given in ‘Results.’ The paper is concluded in ‘Discussion and Conclusion.’

## Related Work

A significant number of papers have been published to deal with the OD detection and segmentation (Haleem et al., 2013). Some papers only perform OD detection while others perform both detection and segmentation. Here we briefly discuss both of the groups.

### Methods for OD detection

Hoover & Goldbaum (2003) use vascular origin to detect the OD center. To detect the vascular convergence point they use a fuzzy convergence and voting type algorithm. Niemeijer, Abràmoff & Van Ginneken (2009) performed vascular segmentation and measure the distance at specific locations with the help of a kNN regressor. The point with lowest distance to the OD is selected as the OD center. Inspired by results from vascular direction methods, (Youssif, Ghalwash & Ghoneim, 2008) used a matched filter to match the direction of blood vessels around the OD area and a vessel direction map is obtained by segmenting vessels. Mendonca et al. (2013) further improve the results by using the entropy of vascular direction to assess the convergence point of vessels. To increase robustness, they constrain the search for maximal entropy to the areas with high intensities. In Lu (2011), a circular transformation is used to capture a circular shape OD and evaluate image variation along multiple radial lines. Pixels with maximum variations are determined, as they can be further used for OD center and boundary localization. Another methodology based on the Radon transformation of overlapping window (Pourreza-Shahri, Tavakoli & Kehtarnavaz, 2014) has achieved 100% success in DRIVE and 96.3% on STARE databases.

### Methods for OD detection and boundary segmentation

In Welfer et al. (2010), a method based on mathematical morphology is proposed to detect and segment the OD in images from DRIVE and DIARETDB1. This work is extended in Welfer, Scharcanski & Marinho (2013) by incorporating a multiscale morphologic approach. Marin et al. (2015) proposed a two step automatic thresholding on a morphologically processed bright enhanced region to get a reduced region of interest, followed by the application of circular Hough transformation (CHT) to get the OD center and OD region. Seo et al. (2004) also use morphological and Canny edge detection filters to segment and detect the OD rim.

Kande, Subbaiah & Savithri (2008) detected the OD by using maximum local variance with 92.53% success rate and geometric active contour model (ACM) for OD segmentation. In Aquino, Gegúndez-Arias & Marín (2010), a template based approach is used for OD segmentation. They applied morphological and edge detection techniques followed by CHT to approximate circular objects. Lupascu, Tegolo & Rosa (2008) used a regression method and texture descriptors for circular OD fitting. An approach based on principal component analysis and mathematical modelling is presented in Morales et al. (2013), which utilizes a generalized distance function, stochastic watershed and geodesic transformations. The result is finally approximated by a circular approximation. Walter et al. (2002) presents a methodology based on watershed transformation and morphological processing. In Hsiao et al. (2012), illumination correction technique was used to detect optic disc. They select high intensity pixels as candidates for OD and among those candidate pixels they select OD pixel as one with the highest variance. For segmentation, the supervised gradient vector flow (SGVF) snake model is used. By extending the SGVF snake in each iteration, contour points get updated and classified based on features. Statistical information and features extracted from trained images were then used for classification. In Joshi, Sivaswamy & Krishnadas (2011), the Chan-Vese model has been extended by introducing image information around a contour point. Inspired by the work proposed in Joshi, Sivaswamy & Krishnadas (2011), the local binary fitting energy ACM Mittapalli & Kande (2016) is proposed to integrate the local image information which includes texture color and intensity for each point of interest. A multi-resolution sliding band filter (SBF) was used in Dashtbozorg, Mendonça & Campilho (2015) for OD segmentation. Super-pixels are employed in Cheng et al. (2013) such that each super-pixel is classified as OD or non-OD. It has been observed that the confluence of vessels in the OD region affects the precision of OD segmentation methods. However, to overcome the influence of the presence of vessels some methods try to eliminate them from image. In this paper, we propose a new approach for automatic OD detection and segmentation which is not influenced by the confluence of vessels in OD area, therefore, no template or vessel map is required in advance.

## The Methodology

This work presents an OD detection and segmentation methodology which is able to detect the OD center without using any template or prior vascular information, an extension to our earlier work (Abdullah & Fraz, 2015). The OD appears as a yellowish structure in retinal fundus images with shape varying from circular to slightly elliptical and has the highest intensity value pixels. However, the presence of brightness artifacts can make the OD merge into the background and lose its brightness. Furthermore, the presence of several pathological structures such as exudates may take the shape of the OD and may have the highest intensity value. The proposed algorithm is based on morphological operations, circular Hough transform and grow-cut algorithm. The morphological operators are used to enhance the optic disc and remove the retinal vasculature and other pathologies. The optic disc center is approximated using the circular Hough transform, and the grow-cut algorithm is employed to precisely segment the optic disc boundary.

### Preprocessing

The variation in image contrast, background illumination and pigmentation is normalized by applying pre-processing operations to the retinal images.

The green channel of an RGB image gives maximum contrast between exudates and the neighboring regions (Fraz et al., 2012a). Therefore, the green channel of RGB images is processed for normalization of contrast and luminosity. A variety of algorithms for contrast and luminosity normalization in retinal images are available in the literature, and these methodologies are either based on subtracting the estimated background from the original image (Fraz et al., 2014) or on dividing the later by the former (Foracchia, Grisan & Ruggeri, 2005; Vázquez et al., 2013). However, our earlier work (Fraz et al., 2014) shows that the results of both methods are similar with no appreciable advantage of one over the other. We have used the subtractive method as it has been reported in our earlier work (Fraz et al., 2014). The background pixel intensities are estimated and the difference between the estimated background and the green channel is computed to produce the normalized image. The background of the retinal image, denoted as “Ibg” is estimated by applying a filtering operation with an arithmetic mean kernel. The size of the filter kernel is not a critical parameter as long as it is large enough to ensure the blurred image contains no visible structures such as optic disc, exudates or blood vessels. In this work, we have used an 89 × 89 pixel kernel. The difference between the estimated background “Ibg” and the morphologically opened image “Iopen” is calculated on pixel basis. Thus, the background normalized image “Inorm” is obtained using:

(1) \begin{eqnarray*}{I}_{\mathrm{norm}}(x,y)={I}_{\mathrm{open}}(x,y)-{I}_{\mathrm{bg}}(x,y).\end{eqnarray*}

Due to different illumination conditions in the acquisition process, there may be significant intensity variations between images. These intensity variations make it difficult to use the best possible technique for all of the images, thus shade corrections were necessary and have been applied. A global linear transformation function is applied to modify the pixel intensities.

**Figure 2:**
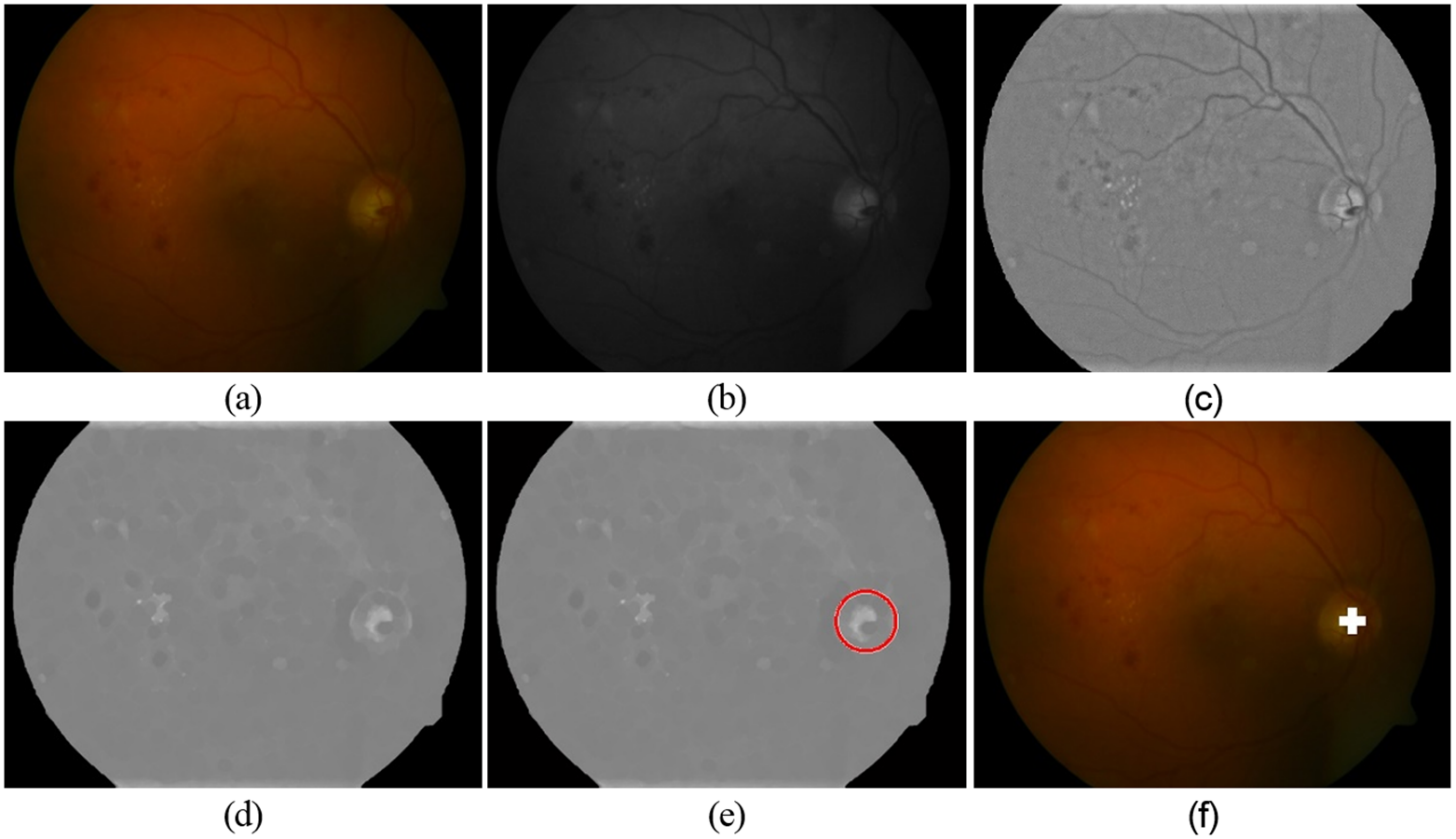
Processing steps for OD detection. (A) RGB retinal image (B) Green Channel image (C) Preprocessed green channel image (D) Blood vessel removed image (E) Circular approximation of optic disc by CHT (F) Detected OD center.

(2) \begin{eqnarray*}{I}_{\mathrm{SC}}(x,y)= \left\{ \begin{array}{@{}ll@{}} \displaystyle 0&\displaystyle \text{if}\hspace*{1em}{I}_{\mathrm{norm}}(x,y)\lt 0\\ \displaystyle 1&\displaystyle \text{if}\hspace*{1em}{I}_{\mathrm{norm}}(x,y)\gt 1\\ \displaystyle {I}_{\mathrm{adjusted}}(x,y)&\displaystyle \text{otherwise} \end{array} \right. \end{eqnarray*}

(3) \begin{eqnarray*}{I}_{\mathrm{adjusted}}(x,y)={I}_{\mathrm{norm}}(x,y)-{\text{IntVal}}_{\mathrm{MaxPixels}}+0.5\end{eqnarray*}

where Isc(*x*, *y*) is the shade corrected image, ${I}_{\mathrm{norm}}(x,y)$ is the background normalized image, ${\text{IntVal}}_{\mathrm{MostPixels}}$ is the intensity value representing the most number of pixels in the normalized image ${I}_{\mathrm{norm}}(x,y)$. Pixels with this intensity value ${\text{IntVal}}_{\mathrm{MostPixels}}$ represent the background (Fraz, Basit & Barman, 2012). This global transformation function normalizes or shade corrects the image by setting the background pixels to 0.5, which can be observed in Fig. 2C.

### Optic disc detection

After pre-processing, the OD appears as the brightest structure in the image with varying size and appearance. The retinal blood vessels originate from the OD and branch out to spread in the retinal image. A morphological closing operation with a disc shaped structuring element is applied to the pre-processed image in order to remove the vasculature from the image. The result is shown in Fig. 2D.

CHT, an extension of Hough transform (HT) (Hough, 1962), is for the detection of circular objects from the image. For the detection of a circle, the HT is based on the equation of circle, defined as

(4) \begin{eqnarray*}({x}_{i}-a)^{2}+({y}_{i}-b)^{2}={r}^{2}\end{eqnarray*}

where, “(a, b)” represents the coordinates of the center of the circle and “r” denotes the radius. In order to increase the performance of CHT we resize all images to a common resolution and search for the bright circles with an experimented selected radius range of 29–50 pixels. To avoid false detection of OD we optimized our system by applying CHT on each image at different sensitivity levels and among circular responses generated by CHT we take only strong circle. Strong circles are the ones that correspond to the OD while the rest are either exudates or misleading regions. The results of intermediary processing steps for OD localization/detection are shown in Fig. 2.

### Optic disc segmentation

In the preprocessed image, the OD area is treated as foreground (fg) and the rest of the retinal image is considered as background (bg). The grow-cut (GC) (Vezhnevets & Konouchine, 2005) algorithm separates the fg from bg using the von Neumann Neighborhood principle (Tommaso & Norman, 1987) and seeded region growing. The detected OD center is chosen as initial seed points for fg area whereas the bg seed points are automatically chosen from rest of image. This algorithm iteratively checks each neighboring pixel and decides its region-wise membership.

The GC algorithm use cellular automata for image modelling. Each image pixel “p” can be represented by a triplet (*l*_*p*_, *θ*_*p*_, *C*_*p*_). Where, “*l*_*p*_” represents the class label of the pixel “*p*” to which it belongs, “*θ*_*p*_” represents the “strength” of the pixel “*p*” which is a measure of the certainty of the pixel “*p*” that should be labelled as “*l*_*p*_”. The label of a pixel whose “strength” = 1 cannot be changed during the algorithm progress, whereas the pixel label whose “strength” < 1 may change during algorithm execution. “*C*_*p*_” represents the pixel greyscale value.

At the initial stage of the algorithm, the triplet for all pixels are set as,

(5) \begin{eqnarray*}{l}_{p}=0,{\theta }_{p}=0,{C}_{p}={\mathrm{RGB}}_{p}\end{eqnarray*}

which means that initially all pixels have undefined labels and zero strength. The aim of the GC algorithm in segmenting the OD is to assign a label to each pixel in the image regarding whether it belongs to OD or to the retinal image background. To start the algorithm, we initialized seeds by setting labels for the optic disc (+1) and non-OD (−1). Once the seeds are initialized, the process keeps on iteratively assigning labels to each pixel in the image until all pixels are labelled. For each pixel *p* and its neighbors *xi* (*i* = 1–8), quantity “*g*” is computed which is monotonous decreasing function where *g* (*xi*) ∈ [0, 1] such that

(6) \begin{eqnarray*}g({x}_{i})= \frac{\parallel {C}_{p}-{C}_{xi}{\parallel }_{2}}{\max {\parallel C\parallel }_{2}} .\end{eqnarray*}

As we were using green channel of the image so ∥*C*_*p*_ − *C*_*xi*_ ∥ ∥*C*_*p*_ − *C*_*xi*_ ∥ is equal to ∥*I*_*p*_ − *I*_*x*_*i*__ ∥, where *I*_*p*_ and *I*_*x*_*i*__ are the intensities of pixels *p* and *xi* respectively. max∥*C* ∥_2_ is equal to 2L - 1, where *L* is the bit depth of the image. Afterwards, the algorithm iteratively compute *λ*(*x*_*i*_) for all pixels *x*_*i*_ which don’t have label “undefined” such that:

(7) \begin{eqnarray*}\lambda ({x}_{i})=g({x}_{i})\theta ({x}_{i})\end{eqnarray*}

**Figure 3:**
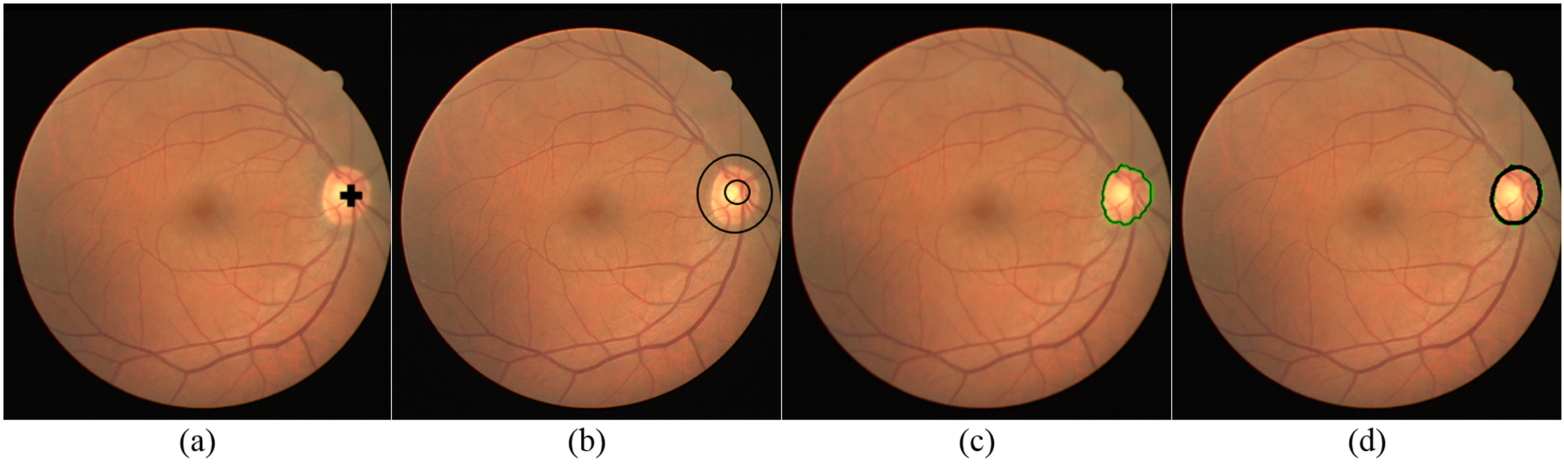
Steps for OD boundary extraction. (A) Original RGB image with detected central point of optic disc, (B) Foreground (optic disc area represented by small circle) selection points and background (non-optic disc region represented by large circle) selection points (C) Result of grow-cut for boundary segmentation, (D) Boundary approximation (in black) of grow-cut.

**Figure 4:**
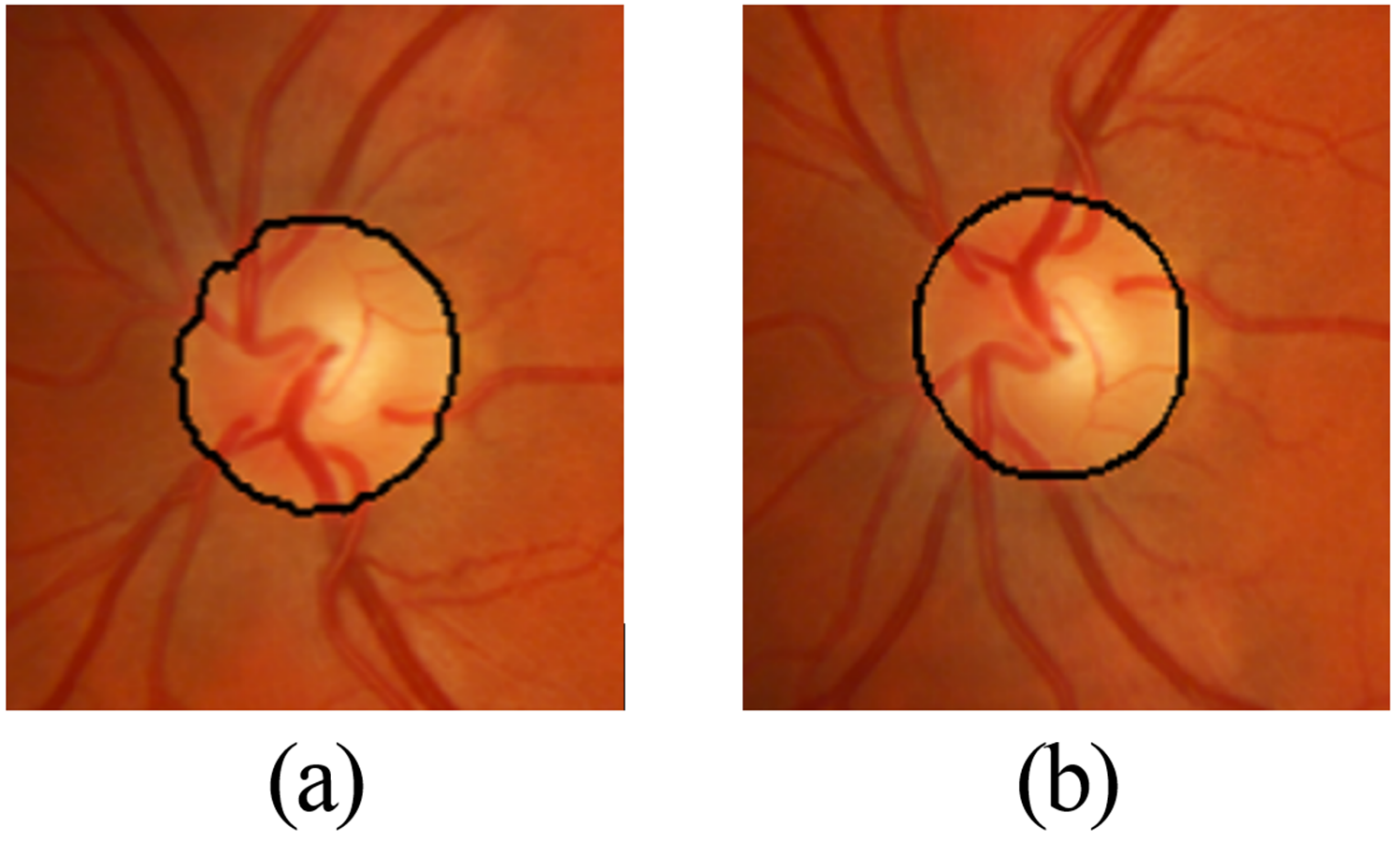
Close up view of (A) grow-cut segmentation; (B) approximation of grow-cut.

If *λ*(*x*_*i*_) > *θ*_*p*_*λ*(*x*_*i*_) > *θ*_*p*_ then a pixel takes the label and strength of *x*_*i*_ otherwise it keeps its own label and “strength.” The algorithm terminates when all the pixels are labeled and the pixel label stops changing. In the end, the segmented OD boundary is approximated to an elliptical shape by using ellipse equation which involves drawing of ellipse outline over the segmented boundary of GC. The processing steps of OD boundary extraction are shown in Fig. 3. The circular approximation of the OD boundary is illustrated in Fig. 4.

## Materials

The proposed methodology is evaluated on five publicly available retinal image databases and one local database.

### DRIVE

Staal et al. (2004) is a publically available database consisting of 40 images with resolution 584 × 565 pixels. Out of these 40 images, seven are pathological, containing pigment epithelium changes, exudates and hemorrhages.

### DIARETDB1

The DIARETDB1 (Kauppi et al., 2007) database comprised 89 fundus images which are obtained with a 50° of FOV using a fundus camera and are in PNG format. These images are of size 1,500 × 1,152 pixels, with 24 bits/pixel.

### CHASEDB1

The CHASEDB1 (Fraz et al., 2012b) database consists of 28 images captured from a Nidek NM 200D camera at 30° FOV. Images are of 1,280 × 960 pixels resolution, which are affected by illumination artifacts and poor contrast.

### DRIONS-DB

The DRIONS (Carmona et al., 2008) database consists of 110 images of 600 × 400 resolution, with 8 bits/pixel. In these 110 images, 50 images contain some sort of defect, such as illumination artifacts, rim blurredness and papillary atrophy, which may hinder the detection and segmentation problem.

### Messidor

The Messidor (Decenciere et al., 2014) database consists of 1,200 retinal fundus images which were captured from 3CCD color video camera on Topcon TRC NW6 non-mydriatic retinograph, with 45° of FOV.

### ONHSD

The ONHSD (Lowell et al., 2004) database consists of 99 fundus images of 640 × 480 resolution. Images were captured from canon CR6 45MNf camera with 45° of FOV. Images were acquired from 50 patients, 19 out of which were diabetic.

### Shifa database

This database belongs to Department of Ophthalmology, Shifa International Hospital Islamabad, Pakistan. A total of 19 images are healthy, while the rest of them have some sort of pathological symptoms and illumination artifacts. The dataset consists of 111 fundus images of 1,936 × 1,296 resolution, acquired with a 45° field of view.

### Ground truths

The OD in all the images from the above mentioned databases is hand labelled by ophthalmic experts from the Armed Forces Institute of Ophthalmology, Rawalpindi, Pakistan and used as ground truths. For 1,200 images in the Messidor database, we have used the ground truths provided by Aquino, Gegúndez-Arias & Marín (2010). The quantitative results are based on comparison of automatic segmented images with these ground truths.

### Quantitative performance measures

The outcome of OD detection and the segmentation process results in the classification of pixels belong to OD region or non-OD region. There are four possibilities for pixel classification, illustrated in Table 1, True Positive (TP), True Negative (TN), False Positive (FP) and false Negative (FN). The first two are the result of mutual agreement between predicted values and actual values while the last two are the result of the wrong prediction. TP is the case when the system predicts the pixel belongs to the OD and is actually an OD pixel in reference to the ground truth image, while in the case of TN both the system and actual ground truth identify a pixel as a non-OD pixel. FP is the case where the system predicts the pixel as an OD pixel when it actually belongs to non-OD region in ground truth, whereas, in the FN case the system predicts a pixel as a non-OD pixel when it actually is an OD pixel.

**Table 1:** Pixel classification.

Real → Predicted ↓	Actual pixel ∈ OD	Actual pixel ∉ OD
System Predicted pixel ∈ OD	TP	FP
System Predicted pixel ∉ OD	FN	TN

**Table 2:** Performance metric for OD segmentation.

Measure	Description
SN	TP/(TP + FN)
SP	TN/(TN + FP)
Acc	(TP + TN)/(TP + FP+ TN + FN)
PPV	TP/(TP + FP)
FDR	FP / (FP + TP)

The metrics used to evaluate the quantitative performance of the proposed methodology are given in Table 2. We used Sensitivity (SN), Specificity (SP), Accuracy (Acc), Positive Predicted Value (PPV), False Discovery Rate (FDR) and Overlap. The overlap metric is defined in (8).

(8) \begin{eqnarray*}\text{Overlap}= \frac{\mathrm{Area}(\text{ground truth}~\cap ~\text{predicted})}{\mathrm{Area}\hspace*{1em}(\text{ground truth}~\cup ~\text{predicted})} .\end{eqnarray*}

Moreover, we have used the DICE similarity index to measure the similarity between the segmented optic disc and the ground truth. The DICE index is a measurement of spatial overlap used widely for comparing segmentation results, with a value ranging from 0 to 1. The DICE coefficient can be defined as two times the volume of the intersection between two segmentations divided by the sum of the volumes of the two segmentations, which is represented in (9).

(9) \begin{eqnarray*}\text{DICE}= \frac{2\ast \text{Area}(\text{ground truth}~\cap ~\text{predicted})}{\text{Area}(\text{ground truth})+\text{Area}(\text{predicted})} \end{eqnarray*}

## Results

### Optic disc detection

The optic disc detection method achieved 100% success rate in DRIVE, DIARETDB1, CHASE_DB1 and Shifa databases, it achieved 99.09% in DRIONS-DB and 99.25% in the Messidor database. Table 3 shows the accuracy of this method for the detection of OD. The comparison of accuracy in localizing OD have been made with other methods reported in literature in Table 4.

**Table 3:** Performance measure of OD detection.

Datasets	Images	OD detected	OD missed	Accuracy
DRIVE	40	40	0	100%
DIARETDB1	89	89	0	100%
CHASE_DB1	28	28	0	100%
DRIONS-DB	110	109	1	99.09%
Messidor	1,200	1,191	9	99.25%
Shifa	111	111	0	100%
ONHSD	90	90	0	100%

**Table 4:** OD localization accuracy comparison with other methods.

Authors	Database	Accuracy
Youssif, Ghalwash & Ghoneim (2008)	DRIVE	98.8%
Niemeijer, Abràmoff & Van Ginneken (2009)	Local database	99.4%
Aquino, Gegúndez-Arias & Marín (2010)	Messidor	99%
Welfer et al. (2010)	DRIVE	100%
	DIARETDB1	97.75%
Lu (2011)	Messidor	98.77%
Zubair, Yamin & Khan (2013)	Messidor	98.65%
Mahfouz & Fahmy (2010)	DRIVE	100%
	DIARETDB1	97.8%
Yu et al. (2012)	Messidor	99%
Saleh et al. (2014)	DRIVE	100%
Yu, Ma & Li (2015)	DRIVE	100%
	DIARETDB1	99.88%
	Messidor	99.67%
Proposed method	DRIVE	100%
	DIARETDB1	100%
	CHASEDB1	100%
	DRIONS-DB	99.09%
	Messidor	99.25%
	ONHSD	100%

**Figure 5:**
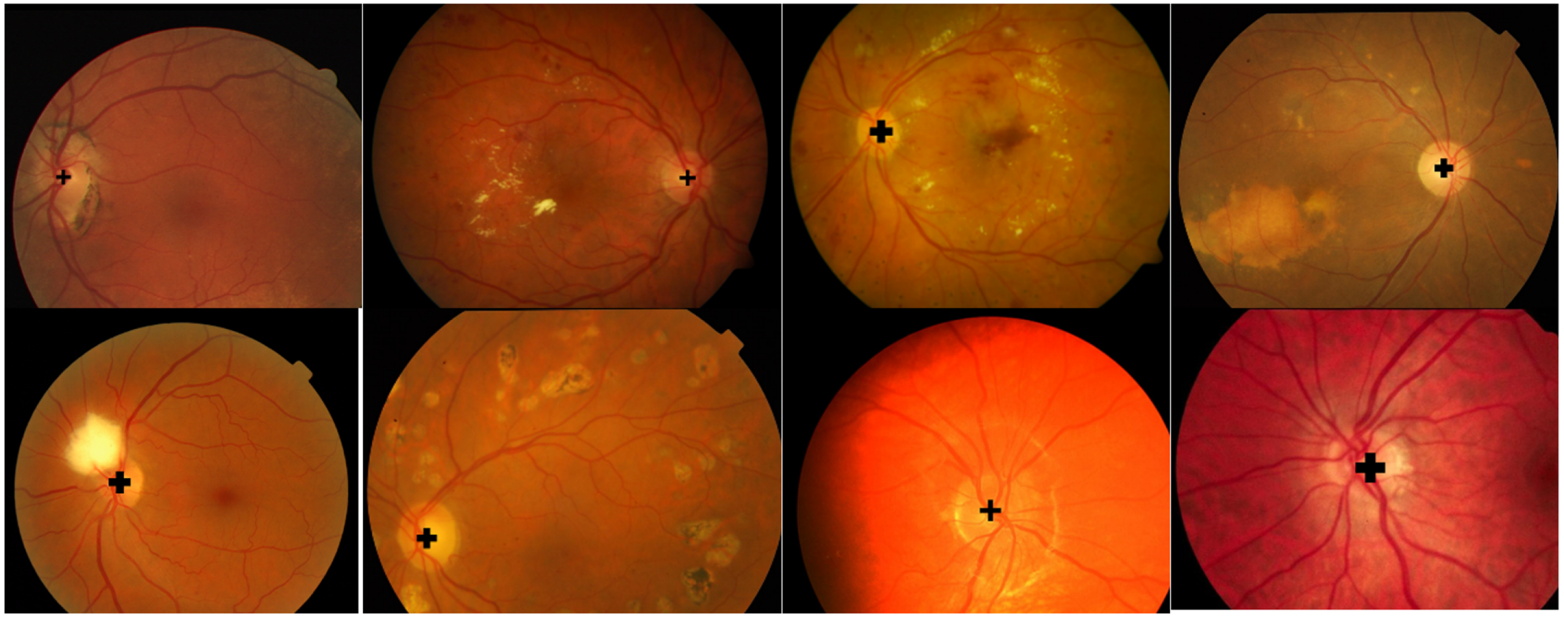
OD detection results. Sorting of images in rows is according to the following order DRIVE, DIARETDB1, Messidore, Shifa, CHASE_DB1 and DRIONS-DB.

Figure 5 shows the results of the OD detection in the DRIVE, DIARETDB1, Shifa, CHASE_DB1 and DRIONS-DB databases. The OD detection method can correctly detect the OD center even in the presence of exudates and other pathologies. Accurate detection of the optic nerve head facilitates the segmentation algorithm to extract the boundary with high precision.

### Optic disc segmentation

The pixel-wise quantitative performance metrics (which are defined in Table 2) are calculated for OD segmentation, based on the comparison of automatic segmented images with the ground truth reference images and are illustrated in Table 5. The methodology is quantitatively evaluated by using an array of performance metrics, which to the limit of our knowledge has not been previously used for evaluating OD segmentation algorithms.

**Table 5:** Performance measures of OD segmentation.

Performance measure	DRIVE	DIARETDB1	CHASE-DB1	Shifa	DRIONS-DB	Messidor	ONHSD
Acc	0.9672	0.9772	0.9579	0.9793	0.9549	0.9989	0.9967
SP	0.9966	0.9984	0.9971	0.9991	0.9966	0.9995	0.9992
SN	0.8187	0.8510	0.8313	0.8015	0.8508	0.8954	0.8857
PPV	0.8728	0.9263	0.9261	0.9493	0.9966	0.9794	0.9619
FDR	0.1271	0.0737	0.0738	0.0506	0.0810	0.020	0.038
DICE	0.8720	0.8910	0.9050	0.8763	0.9102	0.9339	0.9197
Overlap	78.6%	85.1%	83.2%	80.1%	85.1%	87.93%	86.1%
Hausdorff	0.2514	0.1915	0.3174	0.2434	0.2578	0.1627	0.2245

The comparison of the proposed method has been made with other available methods on the basis of average sensitivity, specificity, accuracy, DICE score, overlap, positive predictive value and the time taken to process a single image, as illustrated in Table 6. Results shows that the proposed method provides promising results as compared to other OD segmentation techniques in the literature. The comparison with other methods is made with respect to DRIVE and DIARETDB1, DROINS-DB, Messidor and ONHSD retinal image datasets.

**Table 6:** OD segmentation performance measures comparison with other methods.

Performance measures → Methods↓	Sensitivity	Specificity	Accuracy	DICE score	Overlap %	Predictive value	Average time per image (in s)
**DRIVE Database**							
Sopharak et al. (2008)	0.2104	0.9993	–	–	16.88	0.9334	14.92
Walter et al. (2002)	0.4988	0.9981	–	–	29.32	0.8653	219.6
Seo et al. (2004)	0.5029	0.9983	–	–	31.09	0.843	7.23
Kande, Subbaiah & Savithri (2008)	0.6999	0.9888	–	–	29.66	0.5218	111.7
Sta̧por et al. (2004)	0.7368	0.9920	–	–	33.42	0.6198	43.00
Lupascu, Tegolo & Rosa (2008)	0.7768	0.9968	–	–	30.95	0.88.14	–
Welfer, Scharcanski & Marinho (2013)	0.7357	0.9982	–	–	39.40	0.8876	53.65
Basit & Fraz (2015)	0.8921	0.9921	–	–	61.88	0.6930	–
Morales et al. (2013)	–	–	0.9903	0.8169	–	0.8544	–
Salazar-Gonzalez et al. (2014)	0.7512	0.9684	0.9412	–	–	–	–
Proposed method	0.8188	0.9966	0.9672	0.8720	78.6	0.8728	59.2
**DIARETDB1 Database**							
Sopharak et al. (2008)	0.4603	0.9994	–	–	29.41	0.9593	74.55
Walter et al. (2002)	0.6569	0.9993	–	–	36.97	0.9395	308.5
Seo et al. (2004)	0.6103	0.9987	–	–	35.32	0.8878	15.63
Kande, Subbaiah & Savithri (2008)	0.8808	0.9878	–	–	33.41	0.5448	120.5
Sta̧por et al. (2004)	0.8498	0.9964	–	–	34.08	0.8034	59.72
Lupascu, Tegolo & Rosa (2008)	0.6848	0.9969	–	–	30.95	0.8117	–
Welfer, Scharcanski & Marinho (2013)	0.6341	0.9981	–	–	39.15	0.8704	57.16
Basit & Fraz (2015)	0.7347	0.9944	–	–	54.69	0.7049	–
Morales et al. (2013)	–	–	0.9957	0.893	–	0.9224	–
Proposed method	0.851	0.9984	0.9772	0.891	85.1	0.9263	40.0
**DRIONS-DB**							
Walter et al. (2002)	–	–	–	0.6813	–	–	–
Morales et al. (2013)	–	–	0.9934	0.9084	–	0.9281	–
Proposed Method	0.8508	0.9966	0.9989	0.9102	85.1	0.9794	43.2
**Messidor**							
Morales et al. (2013)	–	–	0.9949	0.8950	–	0.9300	–
Kumar, Pediredla & Seelamantula (2015)	–	–	–	0.8456	–	–	–
Proposed method	0.8954	0.9995	0.9989	0.9339	87.93	0.9794	71.3
**ONHSD**							
Morales et al. (2013)	–	–	0.9941	0.8867	–	0.9310	–
Proposed method	0.8857	0.9992	0.9967	0.9197		0.9619	65.3

**Figure 6:**
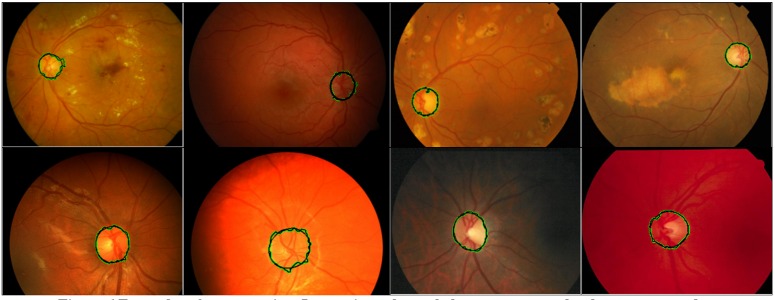
Examples of segmentation. Images in each row belong to separate databases as per order, DIARETDB1, Shifa, CHASE_DB1, and DRIONS-DB. Grow-cut segmentation is represented by a green boundary and its final approximation is represented by a black circle.

The OD segmentation results on these retinal datasets are illustrated in Fig. 6. It can be observed that the proposed methodology can successfully detect and segment the OD in the pathological images as well as in images with non-uniform illumination and uneven background pigmentation from multiple retinal image datasets.

### Robustness of methodology

**Figure 7:**
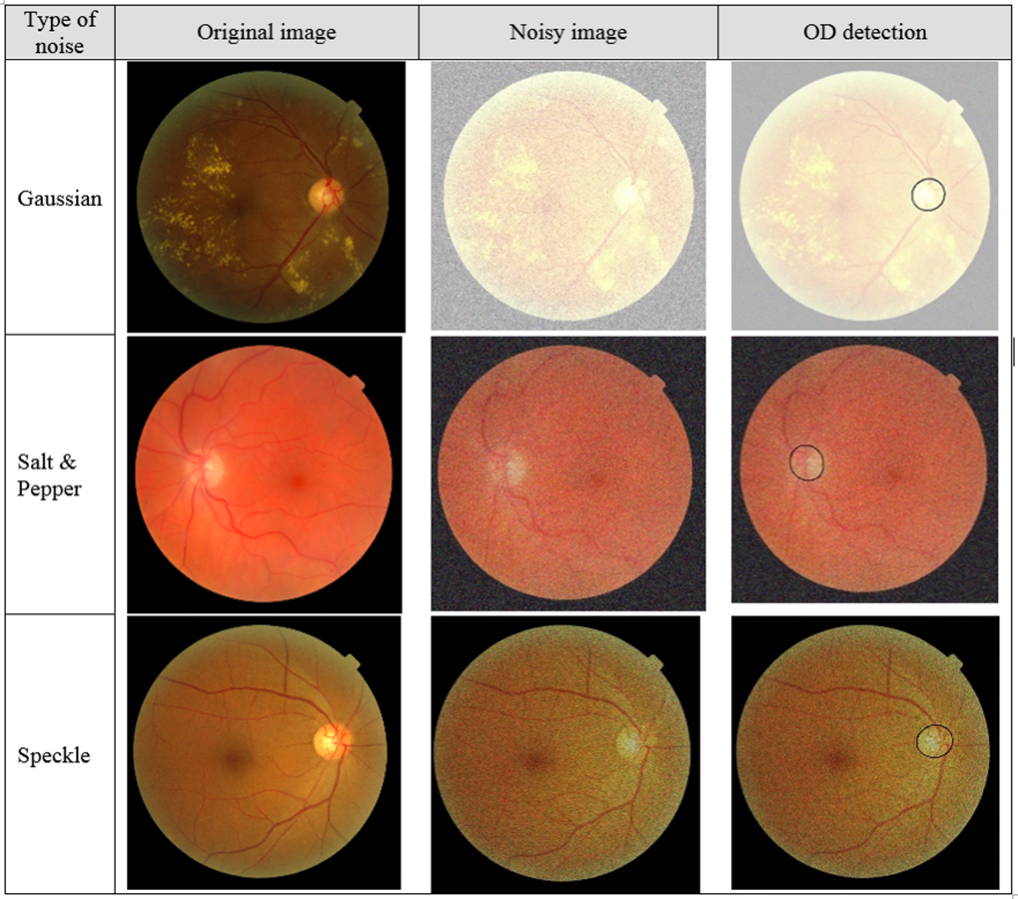
Performance in the noisy images.

The robustness of proposed methodology has been evaluated on the basis of its OD localization and segmentation performance on (1) the noisy images, (2) the images with illumination artefacts, and (3) the images with pathological structures. The retinal images are corrupted with three types of noise models typically found in biomedical images (Gaussian, Salt & Pepper, and speckle noise). It can be observed in Fig. 7, that OD is successfully detected and segmented despite significant deterioration of the retinal images due to the addition of noise.

**Figure 8:**
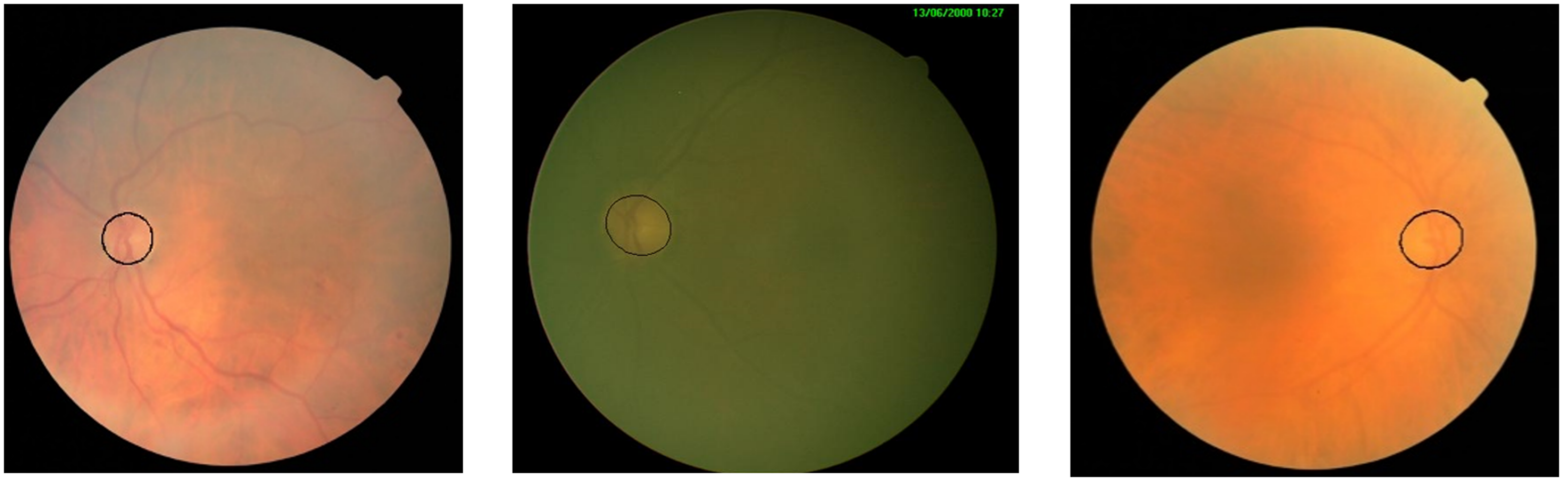
Performance in the poor contrast and uneven illumination.

The second criteria for measuring robustness is the evaluation of against illumination which makes OD detection harder because poor illumination hide the OD in the background, as a result of which segmentation algorithms fails to extract boundary. Figure 8 shows some extreme cases of poor illumination where proposed method successfully localize and segment the OD.

**Figure 9:**
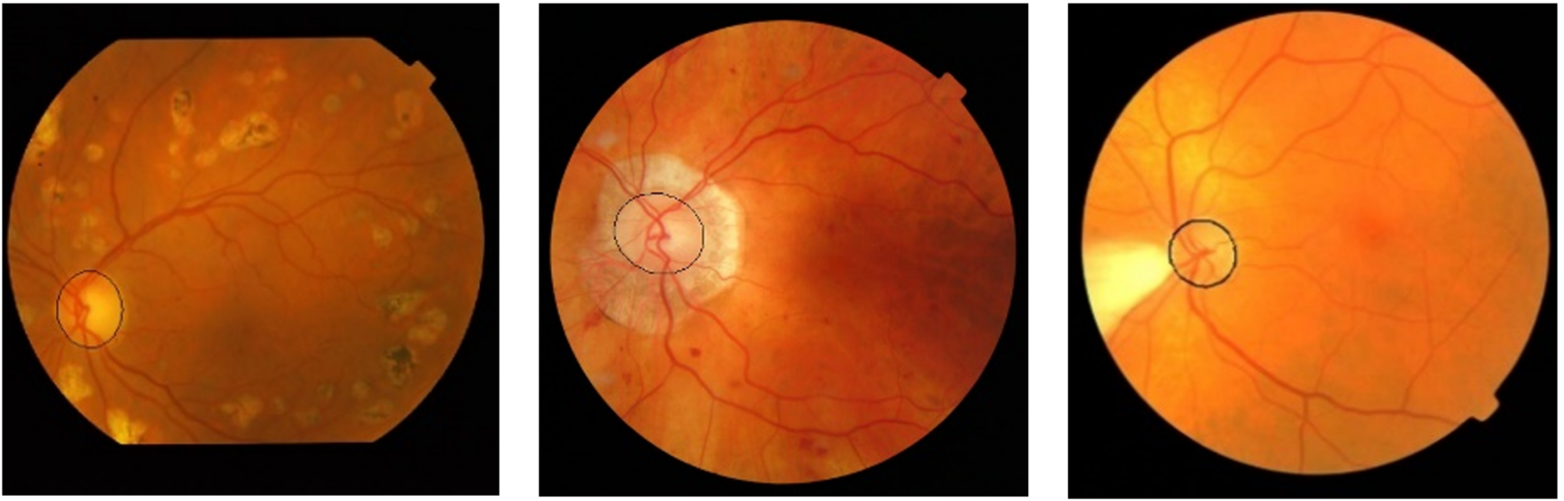
Performance in the presence of pathologies.

Pathologies such as papillary atrophy, exudates, and lesions put a potent threat to accurate segmentation of optic disc because some pathologies may appear in bright or in circular shape and may result in misclassification. While others like papillary atrophy surround the OD and make it difficult to segment. Figure 9 shows the result of proposed algorithm on pathologically affected images.

**Figure 10:**
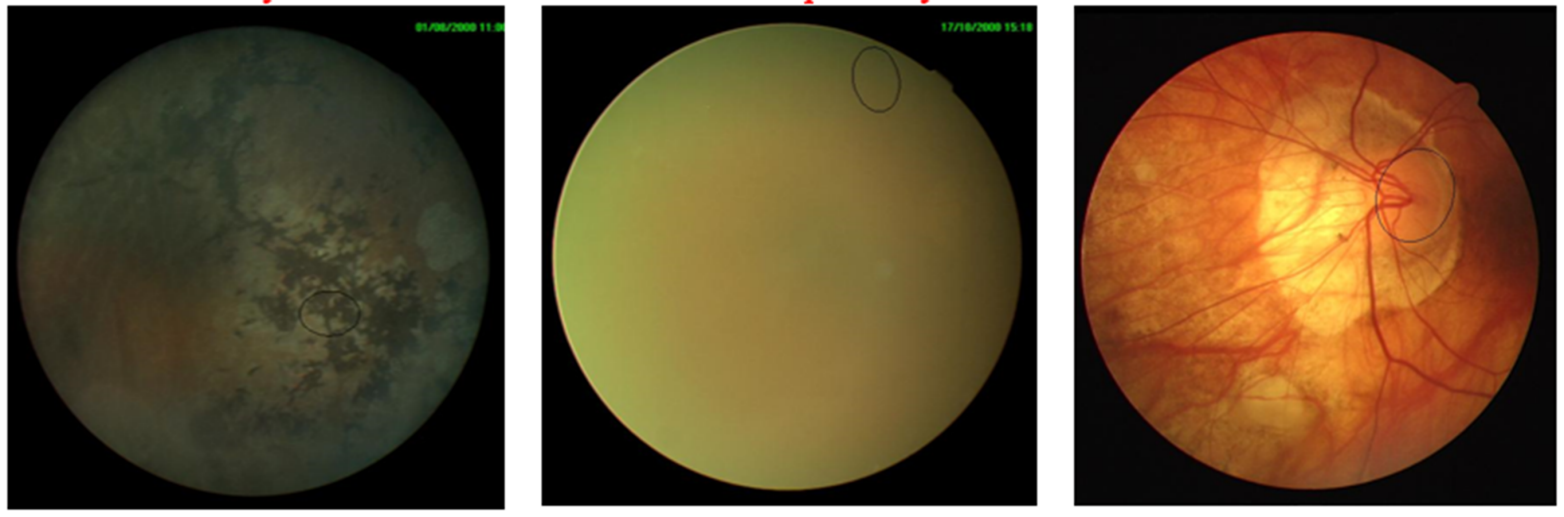
Incorrect OD segmentation in difficult cases of retinal images.

Although the algorithm works well on images where segmentation is hard, in fundus imaging there are sometimes images which are poorly focused or have imaging artefacts which make segmentation a difficult task. Figure 10 shows some extreme cases where the method failed to extract optic disc correctly (e.g., in the first two images the artifacts are so strong that it almost hides OD and leaves a false detection and segmentation, while in the third image pathologies hide the boundary of OD and leave the method to partially detect OD).

## Discussion and Conclusion

Optic disc segmentation is the primary step towards the development of automatic screening systems. The accuracy of the segmentation method improves the correct identification of pathological diseases like glaucoma. Similarly, optic disc detection is the first step towards segmentation and accurate detection would lead to promising segmentation results.

This paper presents a new method for automatic detection and segmentation of the OD in retinal images. Using morphological operations, circular Hough transform and grow-cut algorithm (GC). The GC algorithm has been widely used in many application areas of image segmentation, but has not been applied within the framework of retinal image analysis. To the limit of our knowledge, the GC algorithm has been utilized for the first time in segmenting the OD in retinal images. The method is evaluated on six retinal image datasets exhibiting different morphological characteristics. Experimental evaluation shows that this method is computationally fast in processing, robust to the variation in image contrast and illumination, works well in pathological retinal images and is comparable with state-of-the-art methodologies in terms of quantitative performance metrics. The methodology offers 100% OD detection rate in DRIVE, DIARETDB1, CHASE_DB1, ONHSD, and Shifa databases, and 99.09% success rate in DRIONS-DB1. For the OD segmentation we use the detected OD center point as the seed for the grow-cut algorithm, which then iteratively searches for neighbors of initial seeds and expands the region based on the label and strength of each pixel. The proposed method is able to segment the OD with a better overlap ratio, as compared to other methods available in the literature. We achieved 78.6%, 85.1%, 83.2%, 80.1%, 85.1%, 87.93%, and 86.1% in DRIVE, DIARETDB1, CHASE_DB1, Shifa, DRIONS-DB1, Messidor, and ONHSD databases, respectively. The results of the presented algorithm can be seen online at http://vision.seecs.edu.pk/od/.

OD segmentation results clearly depict the ability of proposed method to segment, even with illumination artifacts when the OD boundary is not clear and in the presence of pathologies like papillary atrophy, which may increase the chances of false positives. The robustness of proposed methodology has been evaluated on the basis of its OD localization and segmentation performance on (1) the noisy images, (2) the images with illumination artefacts, and (3) the images with pathological structures. For evaluation purposes, the retinal images have been corrupted with three types of noises generally found in biomedical images: Gaussian, Salt & pepper, and Speckle noise. The methodology successfully segment the OD despite significant deterioration of the retinal images. Moreover, the algorithms perform well on the images with uneven illumination and the pathological structures.

The work is aimed at contributing to the development of an automatic system for glaucoma detection that is currently under development. Although other published solutions can be used, this work presents higher accuracy, robustness and is tolerant to a vast variety of images which make it suitable for integration with a glaucoma detection system.

We have already developed a fully automated software system named QUARTZ (Fraz et al., 2015), which can extract a number of quantifiable measures from retinal vessel morphology. These measures are analyzed/studied by epidemiologists and other medical/statistical experts in order to evaluate the association of retinal vessel abnormalities with other systemic diseases. In the future, we aim to enhance the aforementioned software system and extend its functionality by incorporating a module for early detection of glaucoma in large population-based screening programs. The proposed method for reliable segmentation of OD can be seen as a first step towards the development of a glaucoma detection module.

## Additional Information and Declarations

### Data Availability

The following information was supplied regarding data availability: The source code and the Optic disc segmentation results are available at: http://vision.seecs.edu.pk/od/.
